# The role of USP36 in ribosome biogenesis and other pathophysiological processes

**DOI:** 10.3389/fmolb.2025.1650908

**Published:** 2025-08-20

**Authors:** Linxin Shao, Mengqi Guo, Qianrui Kou, Ya Guo, Xin Li, Fang Li

**Affiliations:** Yan’an Medical College, Yan’an University, Yan’an, China

**Keywords:** ubiquitin-specific peptidase 36, ribosome biosynthesis, DNA replication stress, hypoxia adaptation, oxidative stress, selective autophagy

## Abstract

Ubiquitination and deubiquitination are common forms of protein post-translational modifications that play crucial roles in the regulation of intracellular homeostasis. As a member of deubiquitination enzyme USP family, USP36 maintains the stability of substrate proteins by mediating their deubiquitination, thereby playing a significant role in various pathophysiological processes. Here we focus on discussing how USP36 participates in regulating ribosome biosynthesis and responds to ribotoxic stress response. Furthermore, this review has elucidated the role of USP36 in regulating DNA replication stress, hypoxia adaptation, oxidative stress, and selective autophagy, as well as the related molecular mechanisms. This review is very helpful for understanding the role of USP36 in pathophysiological process and exploring the possibility of USP36 as a target for disease treatment.

## 1 Introduction

As one of the most critical post-translational modifications, ubiquitination involves the transfer of ubiquitin onto a substrate protein, forming an isopeptide bond, in turn resulting in the alteration of the stability and/or activity of the substrate (Liu F., 2024; Lacoursiere et al., 2022; Dikic and Schulman, 2023). This process is catalyzed by the collaboration of E1 ubiquitin-activating enzymes, E2 ubiquitin-conjugating enzymes, and E3 ubiquitin ligases. Among them, E3 ubiquitin ligases are of crucial importance, as they determine the specificity and extent of ubiquitination of substrate proteins (Zhang, 2025). Conversely, deubiquitination modification is to break the isopeptide bond between ubiquitin and the substrate protein, and deubiquitination enzymes (DUBs) playing a pivotal catalytic role in this cascade reaction (Lim et al., 2013).

There are mainly nine classes of DUBs, including ubiquitin-specific proteases (USPs), ovarian tumor domain proteases (OTUs), ubiquitin C-terminal hydrolases (UCHs), Machado-Joseph domain (or Josephin domain)-containing proteins (MJDs), JAMM/MPN domain metalloproteases (JAMMs), Zinc finger with UFM1-specific peptidase domain protein (ZUFSP/ZUP1), MIU-containing novel DUB family (MINDY), monocyte chemotactic protein-induced protein (MCPIP) and permuted papain fold peptidase of dsDNA viruses and eukaryotes (PPPDE) (Ge, 2022; Ren, 2023). Among these DUBs, the USPs family contains the largest number of members and has been the most extensively studied (Cruz et al., 2021). Enzymes of the USP family can recognize their substrate proteins and deubiquitinate them, maintaining substrate stability, thereby counteracting the effects of E3 ligases on specific cellular substrates. This property has a direct impact on diseases, including cancer (Dewson et al., 2023). The intracellular localization of USPs varies, such as the nucleolus, Golgi body, and endoplasmic reticulum, which helping USPs deubiquitination modification of substrate proteins with different subcellular localization (Clague et al., 2019). Numerous evidences suggest that ubiquitin-specific peptidase 36 (USP36) plays a crucial role in regulating various physiological and pathological processes, including ribosome biosynthesis, ribotoxic stress response, DNA replication stress, hypoxia adaptation, oxidative stress, and selective autophagy (Fang, 2023; Ren et al., 2019; Bhattacharya et al., 2020; Meng et al., 2019). Thus the present article comprehensively reviewed the regulatory functions of USP36 in these pathophysiological processes and the underlying molecular mechanisms.

## 2 USP36 is involved in regulating ribosome biosynthesis and ribosomal stress response

### 2.1 The role of USP36 in ribosome biosynthesis

(1) The role of USP36 in the transcriptional synthesis of rRNA

**FIGURE 1:**
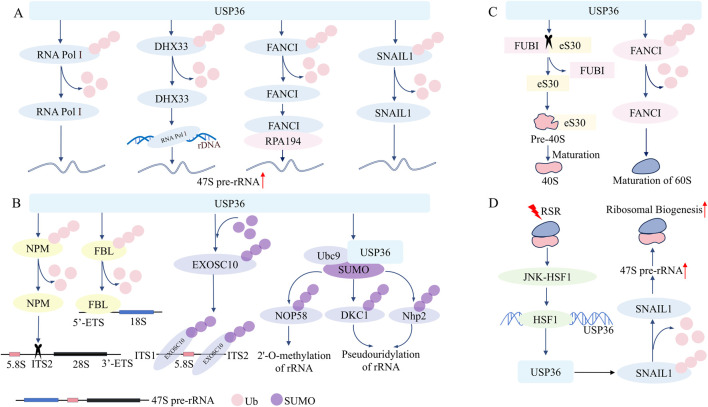
USP36 plays a key role in the regulation of ribosome biosynthesis and ribosomal stress response. **(A)** USP36 enhances the synthesis of 47S pre-rRNA by deubiquitinating RNA Pol Ⅰ, DHX33, FANCI, and SNAIL1. **(B)** USP36 promotes the processing of 47S pre-rRNA by deubiquitinating NPM and FBL, and participates in the maturation and processing of 18S rRNA and 5.8s rRNA by sumoylating EXOSC10 and UBC9. **(C)** USP36 promotes the maturation of the 40S subunit by cleaving FUBI-eS30, and promotes the maturation of the 60S subunit by deubiquitinating FANCI. **(D)** USP36 combats RSR by promoting ribosome biosynthesis.

The synthesis of ribosomal RNA (rRNA) is the initial and rate-limiting step in ribosome biosynthesis (Hori et al., 2023). 47S pre-rRNA serves as the precursor for the three mature rRNAs in eukaryotes: 28S rRNA, 18S rRNA, and 5.8S rRNA. This precursor rRNA is transcribed and synthesized under the catalysis of RNA polymerase I (RNA Pol I), using ribosomal DNA (rDNA) located in the fibrillar center (FC) of the nucleolar region as the template (Bowman et al., 2020). Research has found that USP36 can maintain the stability of RNA PolⅠby reversing its ubiquitin-proteasome degradation, thereby increasing the synthesis rate of 47S pre-rRNA and ultimately participating in regulating the biosynthesis rate of ribosomes (Richardson et al., 2012; Vanden Broeck and Klinge, 2024). Similarly, USP36 can increase the stability of DEAH-box helicase 33 (DHX33) through deubiquitination modificatio, while DHX33 can promote the synthesis of 47S pre-rRNA by enhancing the binding strength between RNA PolⅠand rDNA (Hori et al., 2023; Fraile et al., 2018). These results suggest that USP36 can enhance the protein stability of DHX33 through its deubiquitinating activity, thus promoting 47S pre-rRNA synthesis. USP36 can also maintain the deubiquitinated state of Fanconi anemia complementation group I (FANCI), stabilizing the interaction between FANCI and the large subunit of RNA Pol I (RPA194) in the FC, in turn facilitating the synthesis of 47S pre-rRNA (Sondalle et al., 2019). Additionally, USP36 interacts with snail family transcriptional repressor 1 (SNAIL1) to remove the polyubiquitin chains at positions K146 and K206 of the SNAIL1, achieving the stabilization of SNAIL1 and consequently upregulating the transcriptional synthesis of 47S pre-rRNA (Qin, 2023). Furthermore, it has been reported that knockdown of USP36 can elevate the ubiquitination level of histone H2A, leading to the inhibition of rRNA transcription (Li et al., 2019; Endo et al., 2009a). In summary, USP36 can participate in regulating the transcriptional synthesis of 47S pre-rRNA through mediating the deubiquitination modification of multiple nucleolar proteins such as RNA Pol I, and thereby promote ribosome biogenesis. The production of higher-level nucleolar proteins and the formation of ribosomes can meet the requirements for the high proliferation ability of tumor cells. Given that USP36 is highly expressed in various tumor cells, it is possible that USP36 may have certain carcinogenic potential by regulating ribosome biosynthesis (Figure 1A).

(2) The role of USP36 in the processing and maturation of precursor rRNA

USP36 not only participates in the transcriptional synthesis of rRNA but also in the processing and modification of precursor rRNA (Figure 1B). After synthesis, 47S pre-rRNA undergoes a series of fine regulations to remove the external transcribed spacer (ETS) and internal transcribed spacer (ITS), generating three mature rRNAs: 28S rRNA, 18S rRNA, and 5.8S rRNA (Jiao, 2023; Dorner, 2023). USP36 interacts with nucleophosmin (NPM) and stabilizes NPM through deubiquitination modification. Then NPM recognizes and cleaves specific sequences within the 5.8S-ITS2 region of 47S pre-rRNA through its endoribonuclease activity, thereby facilitating the processing of 47S pre-rRNA (Savkur and Olson, 1998; Endo et al., 2009b; Frazier et al., 2021). Similarly, USP36 colocalizes with fibrillarin (FBL) and mediates the deubiquitination modification of the latter to enhance its stability (Endo et al., 2009a; Endo et al., 2009b). FBL can bind to the 5' ETS of the 47S pre-rRNA precursor through its C-terminal methyltransferase domain, enabling the FBL-associated 47S pre-rRNA to transfer from the FC/dense fibrillar component (DFC) boundary to the DFC for further processing (Yao et al., 2019). Consistent with this, downregulation of USP36 leads to depletion of FBL, accompanied by a reduction in 28S rRNA and 18S rRNA due to impaired processing and maturation of 47S pre-rRNA (Tafforeau et al., 2013).

On the other hand, USP36 is able to regulate the processing and maturation of precursor rRNA through mediating the SUMOylation of certain key nucleolar proteins (Figure 1B). USP36 interacts with the C-terminal Lasso domain of exosome component 10 (EXOSC10) via the basic amino acid extension sequence (amino acids 801–1121) in its C-terminal region, functioning as an E3 SUMO ligase for EXOSC10 (Chen, 2023). By mediating the SUMOylation modification of EXOSC10, USP36 enhances the binding affinity of EXOSC10 to the 18S-ITS1 and 5.8S-ITS2 sequences of 47S pre-rRNA, thereby participating in the regulation of 18S rRNA and 5.8S rRNA maturation (Chen, 2023; Ryu, 2021). Notably, the N-terminal region of USP36 containing the USP domain (amino acids 1–420) can also exert E3 SUMO ligase activity (Ryu, 2021). The N-terminus of USP36 binds simultaneously to Ubc9 (a E2 SUMO-conjugating enzyme) and SUMO, thus mediating the SUMO modification of some protein components (such as Nop58, Nhp2, DKC1) in the small nucleolar ribonucleoproteins complex (Ryu, 2021; Akimoto et al., 2022). Among these, Nop58 is involved in 2'-O-methylation of rRNA, while DKC1 and Nhp2 participate in pseudouridylation, all of which are crucial steps in rRNA maturation (Lui and Lowe, 2013; Wu et al., 2020; Watkins and Bohnsack, 2012). USP36 facilitates the processing and maturation of 47S pre-rRNA. Unlike conventional models, USP36 functions not only as a deubiquitinase but also as an E3 SUMO ligase, catalyzing SUMOylation. Notably, USP36 stabilizes NPM and FBL through deubiquitination. Through SUMOylation of EXOSC10, Nop58, Nhp2 and DKC1, USP36 modulates their functions without changing their mRNA or protein levels (Ryu, 2021).

(3) The role of USP36 in the processing and maturation of ribosome

USP36 is crucial for the maturation of both 40S small subunit and 60S large subunit of the ribosome (Sondalle et al., 2019; van den Heuvel, 2021; O'Dea, 2023). USP36 directly catalyzes the cleavage of the ribosome ubiquitin-like fusion protein FUBI-eS30 into FUBI and eS30. Following this, eS30 integrates into the precursor 40S small subunit, thereby promoting the processing and maturation of the latter. Conversely, if FUBI cannot be effectively removed by USP36, the ribosomal 40S small subunit carrying the FUBI fragment will not be functional in protein translation (van den Heuvel, 2021; O'Dea, 2023). On the other hand, USP36 maintains FANCI in a deubiquitinated state in the nucleolus, ensuring its stability. FANCI is essential for the transcription of precursor rRNA and the processing of the ribosomal 60S large subunit precursor rRNA (Sondalle et al., 2019). In summary, USP36 plays a key role in the processing and maturation of ribosome (Figure 1C).

### 2.2 The role of USP36 in ribotoxic stress response

The ribotoxic stress response (RSR) refers to the response of cells to translation abnormalities, mainly occurring when ribosomes are dysfunctional or overloaded (Snieckute, 2022; Vind et al., 2020). When triple-negative breast cancer cells undergo RSR, the JNK-HSF1 signaling pathway is activated, then USP36 is transcriptionally activated by HSF1. And USP36 stabilizes the SNAIL1 protein through its deubiquitinating activity, thus promoting the precursor rRNA synthesis and ribosome biosynthesis, so as to combat RSR (Qin, 2023) (Figure 1D). These findings suggest that USP36 regulates ribosome biogenesis through multiple mechanisms and tumor initiation and progression by deubiquitinating and SUMOylating various nucleolar proteins. Therefore, targeting USP36 and its regulated mechanisms is expected to be a promising strategy for tumor therapy (Qin, 2023).

## 3 USP36 is involved in regulating DNA replication stress

**FIGURE 2:**
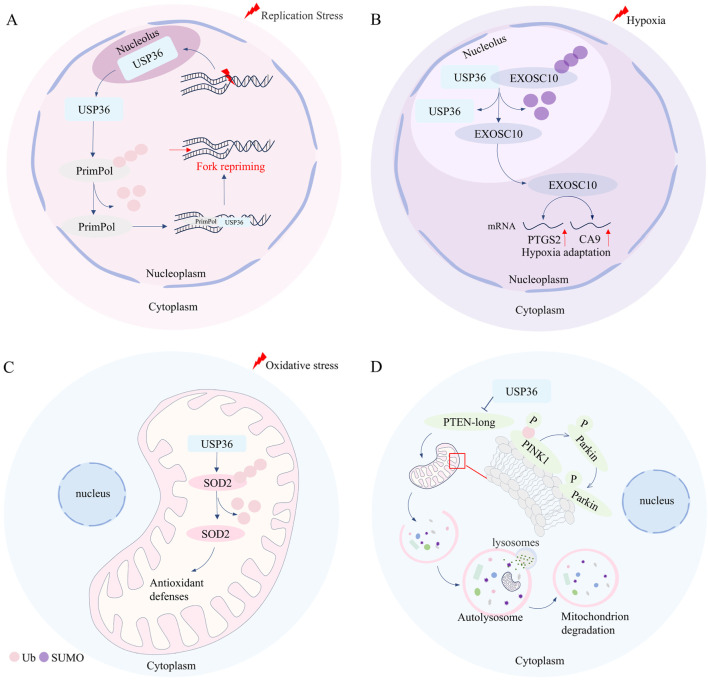
USP36 is involved in the regulation of DNA replication stress, hypoxia adaptation, oxidative stress, and selective autophagy. **(A)** In the condition of DNA replication stress, USP36 facilitates the restart of stalled replication forks by mediating the deubiquitination of PrimPol. **(B)** Under hypoxic conditions, USP36 upregulates the expression of hypoxia-related genes such as PTGS2 and CA9 by mediating the de-SUMOylation of EXOSC10, thereby regulating hypoxic adaptation. **(C)** USP36 can protect cells from mitochondrial oxidative damage through mediating the deubiquitination of SOD2. **(D)** USP36 inhibits the translation of the long form of PTEN, thereby promoting the formation of pSer65-Ub modification on PINK1 on the outer mitochondrial membrane, and marking the damaged mitochondria for autophagic degradation.

DNA replication stress refers to the slowdown or stalling of the progression of replication forks during DNA synthesis, posing a threat to the replication stability of genome (Da Costa, 2023; Berti et al., 2020). DNA replication damage and replication fork stalling can trigger DNA replication stress during DNA replication (Saxena and Zou, 2022). Cells respond to DNA replication stress through various mechanisms, including the reversal of replication forks, the uncoupling of replication forks, and the restart of stalled replication forks (Gaillard et al., 2015). Studies have demonstrated that USP36 is crucial for the effective restart of replication forks (Yan et al., 2020). USP36 is localized in the nucleolus under normal conditions, maintaining the integrity of nucleolar functions. However, when cells experience DNA replication stress, USP36 migrates from the nucleolus to the nucleoplasm and colocalizes with the DNA damage-tolerant polymerase PrimPol at stalled replication forks through its N-terminal USP domain (Yan et al., 2020; Diaz-Talavera, 2022). Yan et al. found that there is a positive correlation between the expression levels of USP36 and PrimPol in ovarian cancer, and USP36 can increase the protein stability of PrimPol through deubiquitination modification, thereby counteracting DNA replication stress (Yan et al., 2020). While knockdown of USP36 significantly increases the sensitivity of ovarian cancer cells to the DNA replication stress inducer hydroxyurea, while restoring PrimPol expression can reverse this phenomenon (Yan et al., 2020). Taken together, these findings suggest that USP36 stabilizes PrimPol through deubiquitination, thus playing a role in counteracting DNA replication stress (Figure 2A) (Yan et al., 2020). High DNA replication stress in cancer cells may lead to genomic instability, and targeting replication stress can help identify new cancer susceptibility loci (Wen, 2025; Da Cos and ta, 2023). Therefore, targeted inhibition of USP36 to block PrimPol-mediated replication fork restart, combined with the use of DNA replication stress pathway inhibitors, may enhance the chemosensitivity of ovarian cancer cells, providing a novel approach for ovarian cancer treatment. Meanwhile, the development of highly selective small-molecule inhibitors targeting USP36 will offer a more specific tool for the precise regulation of DNA replication stress and overcoming tumor chemoresistance.

## 4 USP36 is involved in regulating hypoxia adaptation

The activation of gene transcription mediated by HIF is a crucial mechanism for cells to maintain their survival and function under hypoxic conditions (Yuan, 2024; Batie, 2022). However, USP36 has been reported to be involved in regulating hypoxia adaptation independent of HIF in cervical cancer (Filippopoulou et al., 2020; Chachami et al., 2019; Filippopoulou, 2024). Under normoxic conditions, USP36 and EXOSC10 are co-localized in the nucleolus, where USP36 can function as an E3 SUMO ligase to mediate the SUMOylation of EXOSC10 (Filippopoulou, 2024). However, under hypoxic conditions, EXOSC10 dissociates from USP36 and migrates from the nucleolus to the nucleoplasm along with its deSUMOylation. The deSUMOylation of EXOSC10 upregulates the expression levels of hypoxia-responsive genes (such as PTGS2,CA9, etc.) (Filippopoulou, 2024) (Figure 2B). These findings suggest that USP36 plays a significant regulatory role in hypoxia adaptation.

## 5 USP36 is involved in regulating oxidative stress

Oxidative stress refers to the imbalance of intracellular redox homeostasis, that is, reactive oxygen species are excessively produced, which cannot be offset by the action of antioxidants (Akiyama and Ivanov, 2024). Studies have suggested that USP36 play a role in regulating oxidative stress (Kim et al., 2011) (Figure 2C). USP36 interacts with superoxide dismutase 2 (SOD2) in mitochondria, in turn mediating the deubiquitination modification of SOD2, thereby enhancing the antioxidant capacity of SOD2 (Kim et al., 2011). Further research has demonstrated that the interaction between USP36 and SOD2 is time-dependent and occurs at COP9 signalosome subunit 3 (COPS3) (Kim et al., 2011; Pariano, 2021). The researcher also fund that Anakinra can protect cells from mitochondrial oxidative stress damage by promoting the interaction between SOD2 and USP36-COPS3 (Pariano, 2021). This suggests that targeted regulation of USP36 can enhance SOD2-mediated antioxidant capacity and improve mitochondrial function, providing a potential direction for therapeutic intervention in various diseases, such as Aspergillus fumigatus infection-related pneumonia, mitochondrial dysfunction-associated cardiovascular aging, and age-related neurodegenerative diseases.

## 6 USP36 is involved in regulating the selective autophagy

Autophagy is a process where cells degrade proteins and organelles for reuse, which is crucial for maintaining intracellular homeostasis (Liu, 2023). Based on the selectivity of substrates, autophagy can be broadly classified into two types: selective autophagy and non-selective autophagy (Liu J., 2024; Glick et al., 2010). For selective autophagy, selective autophagy receptors transport specific substrates (such as damaged organelles, aggregated protein or invading bacteria) to autophagosomes for degradation trough binding with LC3 (Vargas, 2023). While, non-selective autophagy is typically induced by factors like rapamycin, nutrient deprivation, or energy starvation (Liu J., 2024). Growing eidence indicates that USP36 plays a vital role in selective autophagy (Geisler et al., 2019; Wang, 2018; Pickrell and Youle, 2015; Ye, 2023) (Figure 2D). USP36 promotes the phosphorylation of Ser65 of ubiquitin (pSer65-Ub) of PINK1 on the mitochondrial outer membrane by inhibiting the translation of the long isoform of phosphatase and tensin homologue (PTEN-long) (Geisler et al., 2019). The activation of PINK1 promotes the recruitment of Parkin from the cytoplasm to the mitochondria, and then ubiquitinates mitochondrial outer membrane protein and marks damaged mitochondria for autophagic degradation (Geisler et al., 2019; Wang, 2018; Pickrell and Youle, 2015). Furthermore, USP36 can also upregulate the protein expression levels of ATG14L and Beclin-1, thereby promoting Parkin-dependent mitophagy during mitosis (Geisler et al., 2019; Ye, 2023). However, it has been reported that USP36 inhibits the selective autophagy in *Drosophila* (Taillebourg et al., 2012). Specifically, USP36 reduces the number of LC3-labeled autophagosomes without affecting the activity of the mTOR pathway (Taillebourg et al., 2012). However, the nuclear localization of USP36 limits the research on its mechanisms of direct action on mitophagy, and further exploration of its downstream targets is required. Additionally, further studies are needed to investigate the applicability of USP36 in regulating selective autophagy across different biological models.

## 7 Conclusion

This review has comprehensively elaborated the role of USP36 in various pathophysiological processes, including ribosome biosynthesis, ribotoxic stress response, DNA replication stress, hypoxic adaptation, oxidative stress, and autophagy activity. USP36 stabilizes RNA Pol I, DHX33, FANCI, SNAIL1, and H2A through its deubiquitinating activity, thereby promoting the synthesis of precursor rRNA. On the other hand, USP36 plays a key role in the processing and maturation of precursor rRNA through mediating the deubiquitination modification of NPM and FBL or mediating the SUMOylation of EXOSC10, Nop58, Nhp2, and DKC1. USP36 further mediates the cleavage of FUBI-eS30 and the deubiquitination of FANCI, promoting the maturation of the ribosome. Additionally, under conditions of ribosomal toxic stress, USP36 can be transcriptionally activated by HSF1 to counteract this stress. It is noteworthy that USP36 also plays a crucial regulatory role in other cellular processes such as DNA replication stress, hypoxic adaptation, oxidative stress, and selective autophagy (Table 1).

**TABLE 1:** The pathophysiological mechanism of USP36 in diseases.

Substrate	Mechanism	Impact	References
RNA Pol Ⅰ	USP36 reverses the degradation of RNA pol I through deubiquitination	Promote the synthesis of 47S Pre-rRNA	Richardson et al. (2012), Vanden Broeck and Klinge (2024)
DHX33	USP36 enhances the stability of the interaction between DHX33 and RNA pol I through deubiquitination of DHX33	Promote the synthesis of 47S Pre-rRNA	Hori et al. (2023), Fraile et al. (2018)
FANCI	USP36 stabilizes the interaction between FANCI and RPA194 through deubiquitination of FANCI	Promote the synthesis of 47S Pre-rRNA and the processing of the 60S large subunit precursor	Sondalle et al. (2019)
SNAIL1	USP36 deubiquitinates the polyubiquitin chains at the K146 and K206 sites of SNAIL1	Promote the synthesis of 47S Pre-rRNA	Qin (2023)
	USP36 is transcriptionally activated by JNK-HSF1, stabilizes SNAIL1 through deubiquitination, and promotes ribosome biogenesis	Resist the RSR	Qin (2023)
NPM	USP36 deubiquitinates NPM and promotes the cleavage of the 5.8S-ITS2 region in 47S pre-rRNA by NPM	Promote the processing of 47S pre-rRNA	Savkur and Olson (1998), Endo et al. (2009b), Frazier et al. (2021)
FBL	USP36 mediates the transfer of 47S pre-rRNA from the FC/DFC boundary to the DFC through deubiquitination of FBL and facilitates its processing	Promote the processing of 47S pre-rRNA	Yao et al. (2019)
EXOSC10	USP36 enhances the binding capacity of EXOSC10 to the 18S-ITS1 and 5.8S-ITS2 sequences through SUMOylation of EXOSC10	Promote the maturation of 18S rRNA and 5.8S rRNA	Chen, 2023; Ryu (2021)
	USP36 dissociates from EXOSC10 and undergoes deSUMOylation, thereby upregulating the expression levels of hypoxia-related genes such as PTGS2 and CA9	Regulate hypoxic adaptation	Filippopoulou (2024)
Ubc9	USP36 mediates the involvement of Nop58 in rRNA 2'-O-methylation and the involvement of DKC1 and NHP2 in pseudouridylation through binding to Ubc9	Participate in rRNA processing and modification	Lui and Lowe (2013), Wu et al. (2020), Watkins and Bohnsack (2012)
FUBI-eS30	USP36 catalyzes the cleavage of FUBI-eS30, enabling eS30 to integrate into the 40S small subunit precursor	Promote the maturation of the 40S small subunit	van den Heuvel (2021), O'Dea (2023)
PrimPol	USP36 colocalizes with PrimPol via its N-terminal USP domain and deubiquitination of the K29 chain of PrimPol	Resist DNA replication stress	Yan et al. (2020), Diaz-Talavera (2022)
SOD2	USP36 deubiquitinates SOD2 and enhances its antioxidant capacity	Regulate oxidative stress	Kim et al., 2011; Pariano (2021)

Research has shown that USP36 promotes tumor progression by deubiquitinating a variety of proteins. USP36 promotes the progression of esophageal squamous cell carcinoma (ESCC) through the Hippo/YAP axis; USP36 enhances the invasiveness of glioblastoma by stabilizing SNAIL2, and USP36 accelerates the progression of hepatocellular carcinoma (HCC) through synergistic action with TP53 (Sun, 2022; Li, 2023; Chang, 2023). The proliferation of tumor cells is highly dependent on ribosome biogenesis, and USP36 has been proven to promote ribosome biogenesis through multiple mechanisms. However, how USP36 regulates ribosome biogenesis in tumor cells has not been fully studied. In addition, specific inhibitors have been developed for other deubiquitinases in the USP family (such as USP1 and USP7), but inhibitors targeting USP36 have not been reported yet (Huang, 2023; Guo, 2024). Therefore, in the future, it is still necessary to deeply study the mechanism of action between USP36 and ribosome biogenesis in tumor cells and explore its potential as a new target for tumor therapy. Meanwhile, the development of specific inhibitors for USP36 should be accelerated to provide new strategies for precise tumor diagnosis and treatment.
